# Challenges in Direct Detection of Flaviviruses: A Review

**DOI:** 10.3390/pathogens12050643

**Published:** 2023-04-26

**Authors:** Bruna de Paula Dias, Camila Cavadas Barbosa, Cyntia Silva Ferreira, Samara Mayra Soares Alves dos Santos, Orlando Alfredo Pineda Arrieta, Wellington Carvalho Malta, Maria Laura Maximiano Dias Gomes, Mariela Alves e Silva, Júlia de Matos Fonseca, Lysandro Pinto Borges, Breno de Mello Silva

**Affiliations:** 1 Department of Biological Sciences, Federal University of Ouro Preto, Ouro Preto 35400-000, Brazil; bdiascmt@gmail.com (B.d.P.D.);; 2 Department of Pharmacy, Federal University of Sergipe, São Cristóvão 9100-000, Brazil

**Keywords:** flaviviruses, arboviruses, diagnosis, direct detection

## Abstract

Arthropods transmit arboviruses via mosquito and tick bites to humans and other animals. The genus flavivirus, which causes diseases, sequelae, and thousands of deaths, mainly in developing and underdeveloped countries, is among the arboviruses of interest to public health. Given the importance of early and accurate diagnosis, this review analyzes the methods of direct detection of flaviviruses, such as reverse transcription loop-mediated isothermal amplification, microfluidics, localized surface plasmon resonance, and surface-enhanced Raman scattering, and presents the advantages, disadvantages, and detection limits identified in studies reported in the literature for each methodology. Among the different methods available, it is essential to balance four fundamental indicators to determine the ideal test: good sensitivity, high specificity, low false positive rate, and rapid results. Among the methods analyzed, reverse transcription loop-mediated isothermal amplification stands out, owing to result availability within a few minutes, with good sensitivity and specificity; in addition, it is the best-characterized methodology.

## 1. Introduction

The genus flavivirus belongs to the family flaviviridae. It comprises positive-sense single-stranded RNA viruses with a genome of ~11 kb [1]. Many of these viruses are transmitted by hematophagous mosquitoes or ticks; therefore, they are called arboviruses (arthropod-borne viruses) [1,2]. Flaviviruses have become a global health problem because of their epidemic potential to cause high morbidity and mortality and infect economically significant animals [3,4,5,6]. Pathogenic flaviviruses include the West Nile virus (WNV), Japanese encephalitis virus (JEV), Yellow fever virus (YFV), Dengue virus (DENV), and Zika virus (ZIKV).

Diseases caused by flaviviruses usually present symptoms similar to those caused by other arboviruses, making it essential to carry out laboratory tests to differentiate viral diseases [7,8]. Diagnostic methods include detection of viral nucleic acids in the acute phase of the disease using techniques based on PCR and/or serological tests during the convalescence phase to detect IgM and IgG antibodies. These conventional serological assays show high cross-reactivity between flaviviruses, owing to similarities in antigenic structures, making differential diagnosis difficult [9,10,11].

PCR-based techniques, especially qPCR, have high sensitivity and specificity. However, some protocols are time-consuming, relatively expensive, and require clinical laboratories with sophisticated instruments and skilled labor, which restricts their use in health facilities [12,13]. Thus, the need for a fast, accurate, easy, and economical method for diagnosing flaviviruses has prompted the development of tools using different technologies for viral detection.

Therefore, we have summarized the tools developed for direct detection of arboviruses of the flavivirus genus in the last decades.

## 2. Conventional Methodologies for Direct Detection of Flavivirus

During the acute phase of viral diseases (1–7 days after the onset of symptoms), some methodologies are ideal for the diagnosis of infection, such as the detection of viral RNA or the detection of the non-structural protein NS1, an essential marker of viremia [14,15]. Viral samples and NS1 protein secreted during viral replication can be found in the blood, plasma, and serum. However, in some cases such as ZIKV infection, viral RNA can also be found in semen, vaginal fluids, saliva, and urine, among other types of biological samples [16].

### 2.1. Molecular Diagnosis

**Figure 1:**
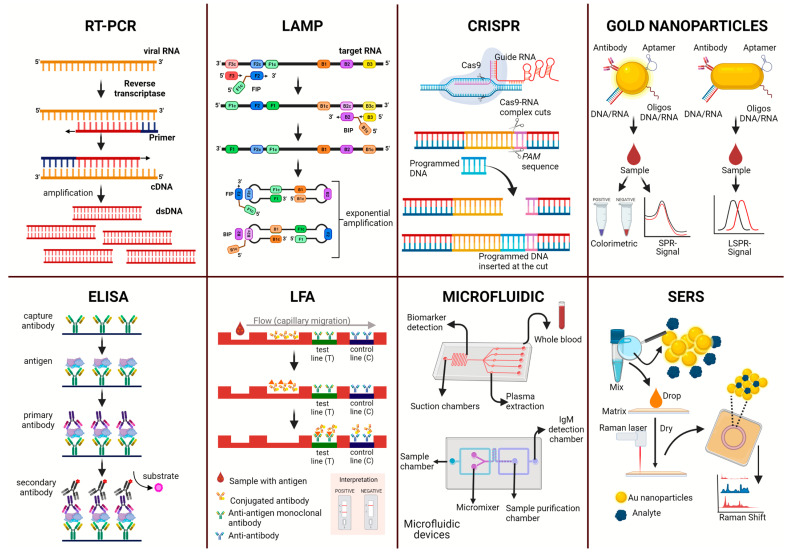
Representation of diagnostic methodologies for the direct diagnosis of arboviruses of the flavivirus genus.

Viral RNA is identified using different types of polymerase chain reaction (PCR), such as reverse transcriptase PCR (RT-PCR), nucleic acid sequence-based amplification (NASBA), or real-time RT-PCR (RT-qPCR). For all PCR variations, viral RNA is extracted from an infected sample after amplification of the genetic material of the virus using the required factors, such as primers, dNTPS, reverse transcriptase, DNA polymerase, and a fluorescent intercalating dye for quantification in the case of RT-qPCR (Figure 1).

One of the main advantages of molecular diagnosis is high sensitivity and specificity. These factors reduce the occurrence of cross-reactions and identify viral pathogens in the early stages of the disease; in addition, these methods are versatile because they can be performed with different biological matrices [16].

Considering the importance of PCR, several protocols have been established to diagnose flavivirus, with several RT-PCR methods already described for the detection of most species of this genus [14]. In addition, combined tests have been proposed, such as RT-qPCR associated with a DNA microarray, which allows rapid (one day) and high-sensitivity identification of mixed infections with different species in field samples (ticks and mosquito vectors) and in human and animal samples [14]. Universal primers for detecting the flavivirus NS5 sequence have also been described, capable of detecting viral genomes with high sensitivity in samples of mosquito cells infected under different conditions with live flavivirus [17].

RT-qPCR generally has a detection limit (LOD) of 5–10 virus copies and is considered the gold standard for diagnosing flavivirus. It differentiates between DENV, ZIKV, and Chikungunya virus (CHIKV). It reduces the possibility of cross-reactions, in addition to identifying the pathogen even in low-level viremia, a fundamental factor for early diagnosis [16]. The pan-flavivirus RT-qPCR assay has also been successfully used in public health surveillance to detect and characterize flavivirus [18]. However, some protocols are time consuming and expensive. They require clinical laboratories with sophisticated instruments and skilled labor, which restricts their use in primary healthcare facilities [12,13].

### 2.2. Serological Diagnosis (NS1 Protein)

The potential clinical use of ELISA to detect the flavivirus nonstructural protein NS1 has been extensively investigated [15,19,20]. This protein is secreted into the blood during viral infection. Therefore, tests have been developed for use in serum, usually with labeled antibodies for NS1 detection. Some of these tests have reduced cross-reactivity and high sensitivity [21,22].

In addition, rapid tests show high performance, as reported for the diagnosis of NS1 in yellow fever, with 100% sensitivity and specificity against DENV types 2 and 3, ZIKV, CHIKV, and Mayaro virus [20]. Furthermore, immunochromatographic methods with NS1/IgM/IgG have also been reported to have high combined performance in the acute phase of dengue, with 91% sensitivity and 96% specificity [23].

Many studies have described homemade protocols that achieve better parameters than commercial tests [22] and rapid antigen-detection assays [24]. The vast majority of studies have focused on the diagnosis of dengue, evaluating cross-reactivity with ZIKV, which is especially important for serological discrimination in endemic regions with co-circulation of these viruses [25]. Therefore, many rapid DENV NS1 detection tests have been developed [26]. These studies indicate that such commercially available assays are less likely to generate false-positive results in clinical samples in areas in which several flaviviruses coexist [24]. Recent studies have also shown good sensitivity (82%) and specificity (93%) for NS1-based ELISA detection of dengue infections without cross-reactivity with ZIKV [27]. Furthermore, dengue NS1 capture assays for early and differential diagnosis also showed excellent parameters in the context of co-circulation with ZIKV (specificity of 99.32% and accuracy of 92.43%) in different samples (serum, plasma, and urine) [28].

## 3. New Methodologies for Direct Detection of Flavivirus

### 3.1. RT-LAMP

Among the existing nucleic acid amplification methodologies, Reverse transcription loop-mediated isothermal amplification (RT-LAMP) stands out as a fast, simple, sensitive, and specific technique [29,30,31]. With the RT-LAMP technique, gene amplification and detection can be performed in a single step. In this assay, the sample, primers, and Bst DNA polymerase are incubated in a single tube at constant temperature. The results can be monitored in real time by measuring the turbidity of the sample with a photometer [30] or the changing color, either with the naked eye [32,33,34] or with a smartphone [35]. Furthermore, because amplification occurs under isothermal conditions and does not require a thermocycler, it can be performed in a simple heat block or water bath. Therefore, this technique can be applied using simpler equipment, which allows the application of RT-LAMP in healthcare locations with limited resources.

To prevent the expansion and propagation of ZIKV, several authors have developed methodologies to detect this virus based on the RT-LAMP technique. Wang et al. [36] designed an RT-LAMP assay for ZIKV detection using a portable battery-powered metal bath to perform all sample processing and testing steps. The results were analyzed with the naked eye within one hour. The assay had a limit of detection (LOD) of 4000 copies/mL for standard ZIKV RNA and 4 PFU/mL for simulated clinical virus samples. In endogenously infected human (urine and serum) and mosquito samples, LODs of approximately one copy of the genome were obtained using RT-LAMP for ZIKV [37].

Silva et al. sought to validate RT-LAMP for ZIKV detection in mosquito samples. RT-LAMP detected a wide range of viral concentrations (from 10^5^ to 10^−5^ PFU) in the *Aedes aegypti* crude lysate. The LOD at 95% probability for each RT-LAMP was -2.98 log10 PFU of ZIKV (~1/1000 PFU) with a confidence interval of −3.62 to −1.64. In samples of experimentally and naturally infected *A*. *aegypti* and *Culex quinquefasciatus*, RT-LAMP showed a sensitivity of 100%, specificity of 91.18%, and overall precision of 95.24% for RT-qPCR, showing a better analytical sensitivity than that of RT-qPCR [13].

The combination of RT-LAMP with magnetic streptavidin nanoparticles to detect ZIKV oligonucleotides was also efficient. In this system, the proposed biosensor achieved an LOD of 1 attomolar synthetic ZIKV oligonucleotide with a total assay time of 27 min. The same sensitivity was observed when performing the test in serum samples [38]. In a portable battery-powered device, the RT-LAMP assay for detecting ZIKV detected viral RNA at 14.5 TCID50 in virus-enriched serum or urine samples within 15 min. Using standard RNA, the detection limit of the assay was estimated to be 10 copies per reaction [39].

Priye et al. [35] developed a system in which they coupled RT-LAMP with the technique of quenching unincorporated amplification signal reporters (QUASR). The reactions were performed in a “LAMP box” complemented with a detection system based on the analysis of images generated using a smartphone. In the initial assays, positive amplification was detectable within 10–15 min, detecting sample RNA concentrations of 10^5^–10^2^ PFU equivalent/mL (10^4^–10^1^ copies/rxn). By performing RT-LAMP in the “LAMP box,” it was possible to detect ZIKV at amounts below 100 PFU/mL directly from a matrix of human body fluids in clinical samples at realistic concentrations.

For DENV, Zhou et al. [33] established a new RT-LAMP method with an LOD of 74, 252, 78, and 35 copies of viral RNA per reaction for DENV-1, DENV-2, DENV-3, and DENV-4, respectively, within 50 min. The RT-LAMP assay developed by Neeraja et al. [40] aimed to carry out the detection and serotyping of DENV infection by targeting specific regions of the NS1 gene using a real-time fluorometer (Genie II fluorometer). The color change could be observed within minutes and had a detection limit of 100 copies for DEN-1 and DEN-2, and ten copies for DEN-3 and DEN-4. In the assay reported by Lau et al. [32], the LOD was 10 RNA copies for all DENV serotypes, with a specificity and sensitivity of 100%.

In the RT-LAMP assays to detect the YFV, it was possible to obtain an LOD of 0.29 PFU/mL [41] and 19 PFU/mL [42]. For the Japanese encephalitis virus, it was possible to obtain an LOD of 2.34 copies/µL [43], 5 pg of RNA [44], and 12 copies/µL [45]. The LOD for the West Nile virus was 0.1 PFU/mL [46].

### 3.2. CRISPR

Clustered regularly interspaced short palindromic repeats (CRISPR) is a guide RNA (sgRNA) that, associated with proteins (Cas), can recognize, cleave and even insert DNA/RNA sequences into in vitro and in vivo models. Diagnostic systems based on CRISPR/Cas have high sensitivity, specificity, and low cost [47].

Gootenberg et al. [48] demonstrated the use of CRISPR for detecting DENV and ZIKV. The SHERLOCK system (specific high-sensitivity enzymatic reporter unlocking) uses an isothermal amplification through recombinase polymerase amplification (RPA), followed by detection and cleavage of the target sequence by CRISPR/Cas13a. When activated by the presence of viral genetic material, Cas13a promotes collateral cleavage of the marker, releasing a fluorescent signal. The assays had a detection limit of approximately 2 attomoles in standardized virus samples within 22 min. The assay time was 3 h for human serum and urine samples containing the ZIKV. To improve the technique for use in the field, the reagents were lyophilized and rehydrated immediately on paper. The paper-based assay had a detection limit of 20 fmol of non-amplified ssRNA.

Gootenberg et al. [49] improved the second version of the SHERLOCK system, using a new protein, LwaCas13a, associated with another CRISPR component, Csm6. It was possible to increase the sensitivity of the signal and develop multiple reactions with up to four channels in the same CRISPR reaction, making it possible to differentiate similar viruses in a single reaction, presenting a quantitative result and the possibility of carrying SHERLOCKv2 for reading on lateral flow strips. ZIKV or DENV ssRNA was detected in less than 90 min, with sensitivities down to the attomolar range.

Myhrvold et al. [50] developed HUDSON (heating unextracted diagnostic samples to obliterate nucleases), a method to lyse viral particles and inactivate the high levels of RNases found in body fluids using heat and chemical reduction. HUDSON combined with SHERLOCK allowed the detection of ZIKV RNA from infectious particles at 90 attomolar concentration (45 cp/µL) in whole blood or serum, 0.9 attomolar (~1 cp/µL) in saliva, and 20 attomolar (10 cp/µL) in urine, with a total response time of <2 h with fluorescent and colorimetric readings. Furthermore, the authors designed a panel using DENV-specific RPA primers and serotype-specific crRNAs to distinguish between DENV 1–4 serotypes with <3.2% off-target fluorescence. The low level of off-target fluorescence allowed for 100% specificity in differentiating between the DENV serotypes.

### 3.3. Microfluidics

Microfluidics can be described as the science and technology of systems that process or manipulate fluids in small volumes (10^−9^ to 10^−18^ L) using microchannels [51]. Microfluidic devices offer significant sample and reagent reduction, lower energy consumption, speed, portability, and simplicity of use [52,53,54,55]. Furthermore, depending on the application, the devices can be manufactured from different materials such as elastomeric polymers, glass, silicone, and paper [56].

For several reasons, analytical devices based on microfluidic paper (μPADs) are more advantageous than conventional microfluidic analytical devices. First, paper is thin and light, which facilitates stacking, storage, and transport. Second, paper is readily available worldwide and inexpensive. Third, paper absorbs aqueous fluids, making passive fluid transport without active pumping practical for μPADs. Fourth, paper is compatible with biological samples. Fifth, paper can be chemically modified to incorporate various functional groups that can be covalently linked to proteins, DNA, or small molecules. Finally, paper is biodegradable and recyclable [57,58,59].

Prabowo et al. [60] used a wax-printed µPAD to detect DENV NS1 protein (DEN-NS1-PAD). Assays were performed using buffer, culture medium, and serum samples. The color change related to the detection of DENV NS1 protein was analyzed with the naked eye, using a scanner and a smartphone camera, and reached an LOD of 200, 46.7, and 74.8 ng mL^−1^, respectively. The determination of NS1 using DEN-NS1-PAD took only 20–30 min by the naked eye and 30–60 min by image processing.

Bedin et al. [58] developed and evaluated a wax-printed μPAD for the detection of NS1 from DENV and ZIKV. Assays were performed using whole blood and plasma samples enriched with recombinant NS1 protein and tested in simplex and multiplex formats. For ZIKV NS1 and DENV serotypes 1, 2, and 3, the LOD in plasma were estimated to be 10 ng/mL and 20 ng/mL blood. The results were obtained in less than 8 min. In this study, the authors also tested plasma samples from patients with acute DENV, using the μPAD. The results showed that all NS1-positive patients were detected as positive by μPAD, demonstrating its effectiveness in detecting patients with acute dengue. It was also possible to specifically detect Zika and/or dengue NS1 proteins in a multiplexed μPAD.

Although the most common method for manufacturing paper-based microchips is wax printing, hybrid materials are interesting because they combine the advantages of both materials. Draz et al. [61] developed a hybrid paper-plastic microchip (PPMC) to identify ZIKV by electrically detecting viral lysates. The LOD of the proposed microchip was 10^2^ particles/µL ZIKV in phosphate-buffered saline.

In the study by Yuzon et al. [62], the microfluidic hybrid chip was made of a nitrocellulose (NC) membrane, cellulose acetate (CA) film, and polymethyl methacrylate (PMMA) sheet fabricated using simple wax printing, stacking, baking, and binding procedures. The fabricated chip was designed to be integrated with the LFIA technique and to take advantage of the microfluidic mechanism for the detection of the NS1 DENV antigen. The assay time was less than 2 min, and the LOD was determined to be 84.66 ng/mL.

Combining these systems, Lee et al. [63] developed a μRT-PCR system to detect DENV. First, the sample was purified/enriched in the proposed system using superparamagnetic granules. The virus was subjected to thermolysis, RNA extraction, and RT-PCR on a single chip. The detection limits were approximately 10–10^2^ PFU/mL, and the test time was 3 h and 22 min.

Sharma et al. [12] performed an automated microfluidic chip-based LAMP assay that combined the isolation, purification, and amplification steps on the same platform. It allowed the visual detection of 10^2^ copies/mL of ZIKV in human plasma within 40 min. Detection of ZIKV via a wax-printed paper microfluidic chip using RT-LAMP has also been described [64]. The trial produced successful visible color changes within 15 min, which were quantified using smartphone imaging. The LOD was one copy/μL, and amplification was performed on complex samples (tap water, urine, and 10% diluted plasma) [64]. On the other hand, a microfluidic platform for the detection of ZIKV in an RT-LAMP assay with real-time visual analysis performed by smartphone images could detect up to 10 PFU/reaction, which corresponds to 1.56 × 10^5^ PFU/mL of virus in infected blood, and in less than 35 min with minimal sample processing [65].

Lo et al. [66] combined an RT-LAMP assay with a paper-based diagnostic platform for DENV-2 detection. U-safe fluorescent probes were used for the assay to detect 0.75-fold diluted RT-LAMP products that were reverse-transcribed and amplified from a virus concentration of 6 x 10^3^ PFU/mL. The total diagnostic time was 100 min.

### 3.4. Nanotechnology-LSPR

The application of nanoparticles in diagnostics has shown promise, owing to their physicochemical properties and large surface area and volume, which contribute to increased sensor sensitivity [67,68]. Furthermore, given the diverse nanomaterials used to create nano-biosensors, Au and Ag nanoparticles have been highlighted for their distinct optical characteristics used for developing plasmonic sensors [69].

Among the types of plasmonic sensors, localized surface plasmon resonance (LSPR) sensitivities are lower by orders of magnitude than those of other sensors [70]. The LSPR technique measures the changes in the refractive index due to the binding process of molecules on the surface of the nanoparticles. When functionalized, they can detect RNA, DNA, and viral proteins [68,69].

These refractive index changes can be monitored in real time by reading the LSPR band in a spectrophotometer or by changing the color of the sample solution with the naked eye, owing to the aggregation or dispersion effect of the nanoparticles. Specifically, gold nanoparticles (AuNPs) change the color of the solution from red to blue/purple and silver nanoparticles (AgNPs) from yellow to orange [71].

The colorimetric properties of AuNPs and AgNPs can be adjusted by varying their shape and size, which allows for multiplexed analysis. Therefore, Yen et al. [72] conjugated generate solutions of different colors when detecting the NS1 protein of DENV and YFV. The multiplexed detection could be differentiated with the naked eye as orange (YFV) and green (DENV), and an LOD of 150 ng/mL was obtained.

Awan et al. [73] used spherical AgNPs to manufacture an immunosensor to detect the dengue NS1 protein. The well-defined and clear electrochemical signals generated by AgNPs enabled the specific detection of NS1. A proportional increase in the faradic current for the oxidation of AgNPs was observed with an increase in the NS1 protein concentration. The proposed immunosensor achieved a detection limit of 0.5 ng/mL.

Carter et al. [74] used AuNPs to associate DNAzyme activation (DDZ) with the salt-induced aggregation of nanoparticles to perform colorimetric detection of DENV RNA. The system resulted in rapid aggregation of AuNPs, changing the color of the solution from red to a lighter color in as little as 5 min. The LOD achieved was 1 × 10^1^ DENV TCID_50_ units, which corresponds to 0.06 nM of DENV RNA per reaction.

To create new colorimetric diagnoses based on aptamers, Bosak et al. [75] developed and tested aptamers and Apt-AuNPs that specifically detect proteins in mosquito saliva (*Ae*. *aegypti* and *Ae*. *albopictus*), as well as novel aptamers and Apt-AuNPs against ZIKV. In vitro tests in *Ae*. *aegypti* showed visible colorimetric detection with an LOD of 10 ng of salivary gland extract, and 10^5^ PFU/mL of spectrophotometrically detectable active ZIKV.

Basso et al. modified the surface of AuNPs with anti-dengue antibodies by exploring the plasmonic band of the AuNPs. The created immunosensor was able to identify DENV in less than 5 min, and obtained an LOD of 10^7^ TCID_50_ [76]. Farooq et al. [77] presented a platform for LSPR sensors with AuNPs functionalized for the identification of NS1 antigens of DENV. The assay results showed an LOD of 0.07 μg/mL (1.50 nM) of NS1 when the nanoparticles were incubated for 1 h.

### 3.5. Surface-Enhanced Raman Scattering (SERS)

Raman scattering is an inelastic process in which incident photons gain or lose energy owing to the vibrational and rotational motion of the analyte molecule [78]. As a result of these movements, distinct spectra of molecules are generated, providing a “fingerprint” of the analyte. The use of surface-enhanced Raman scattering (SERS) appears as an amplifier of Raman intensity, in the order of 10^6^ and 10^8^ fold, enabling its use in biological materials [78,79,80].

The application of the SERS effect in biosensors is advantageous due to several factors. First, it enables direct analyte identification. Second, it has high sensitivity. Third, it eliminates expensive reagents or associated time-consuming sample preparation steps. Fourth, SERS labels are not susceptible to photobleaching. Finally, it has multiplex capability, as spectral widths are 10–100 times narrower than fluorescence labels, thus minimizing the overlap between different labels [78,79,80]. In addition, portable equipment is already available for measuring the spectra; therefore, this methodology can be used to perform diagnoses outside the laboratory.

Given the potential advantages of the SERS approach, Ngo et al. [81] reported a DNA-on-chip bioassay system using SERS detection to diagnose DENV. This system detected approximately 6 attomoles of DENV4 ssDNA. Zhang et al. [82] proposed the detection of the WNV target sequence using SERS spectra through magnetic separation of the hybridization of the target sequence of the virus with paramagnetic nanoparticles and gold nanoparticles conjugated with a Raman reporter tag. Hybridization provided a spectrum of SERS signatures with a 10 picomolar detection limit for the target sequence. The assay required approximately 1 h to perform, from the assembly of the fabricated reporter GNPs to the acquisition of SERS spectra.

Another assay using SERS based on magnetic capture combined with the conjugation of polyclonal antibodies specific for WNV protein E in paramagnetic nanoparticles (PMPs) and Raman reporter-coated Au nanoparticles (GNPs) was proposed by Neng et al. [83]. The GNP/antigen/PMP complex was magnetically concentrated. The generated Raman spectra provided a detection limit of ~5 femtogram/mL for assays performed in phosphate-buffered saline (PBS), and ~25 picogram/mL for assays with PBS enriched with fetal bovine serum.

DENV and WNV envelope proteins were detected using a SERS probe based on bio-conjugated gold nanoparticles (AuNP). The constructed probe proved to be a sensitive fingerprint detection tool, capable of detecting 10 PFU/mL of virus in less than 30 min [84].

## 4. Limitations

Although LAMP has several advantages, assay constructs are more labor-intensive than PCR assays. The design of the primers is a focus of attention, as incompatibilities between the primers can reduce amplification efficiency [33,85]. In addition, cross-contamination may occur [31,86], and according to Sahoo et al. [85], multiplexing assays are less developed than PCR.

In CRISPR-based detection, there is a possibility of binding and non-specific cleavage (off-target effect); however, the inclusion of bioinformatic analyses to select better sgRNAs can reduce these effects [47,87].

μPADs, however, still need to be tested on an industrial scale. In addition, it is essential to evaluate the transport and storage processes due to the reagents used in the devices, such as antigens and antibodies. These tests may depend on particular conditions and the development of multiplexing capability [59,88].

Regarding LSPR nano-biosensors, more studies need to be performed to demonstrate the efficiency of these sensors in real samples (such as patient serum and naturally infected mosquitoes, among others), as non-specific binding can cause interference in detection [69]. Furthermore, the bulky and costly nature of the detection device is a disadvantage of the technique, although it can be circumvented by colorimetric and electrochemical approaches that do not require expensive and complex instrumentation to obtain results [69,72,74].

Similarly, to nano-biosensors, biosensors based on SERS detection still need to demonstrate efficiency in real samples. The analysis using most of these assays takes approximately 1 h, which can be a disadvantage, as other methodologies can detect the target in a matter of minutes. Furthermore, some authors have pointed out that the instrumentation needed to measure Raman scattering is not commonly found in laboratories based on molecular diagnostics, which makes widespread adoption of this technique difficult [89].

The LOD and the advantages of each of the methods for the direct detection of flaviviruses have been presented (Table 1). However, there are still limitations to overcome in these technologies to find the optimal test.

## 5. Conclusions

In conclusion, we described diverse technologies that have been developed for the direct detection of arboviruses of the flavivirus genus. New technologies are intended to facilitate diagnosis through portable, simple, and low-cost techniques, improving and maintaining the sensitivity and specificity of the currently available methods.

Despite advances in these technologies, there is still a need to test complex samples to assess the effectiveness of implementation in clinical practice. Furthermore, considering that the absence of a timely and correct diagnosis can worsen the diseases, it is essential to perform diagnostic testing during the optimal period to maximize the advantages of the test. Therefore, it is essential to study reproduction and detection methods to control the spread of vectors and to reduce the number of arboviruses.

This study is extremely relevant to illustrate the different methods used in the diagnosis of flaviviruses and to assist healthcare staff in the best choice of test based on the purpose and available resources. We noted that the tests evolved over time, and the RT-LAMP test proved to be ideal and superior to the others, as it provided the results within a few minutes, had good sensitivity and accuracy, and did not require expensive and complex instrumentation.

## Figures and Tables

**Table 1:** Summary of flavivirus diagnostic platforms.

Method	Detect	Detection Limit (LOD)	Time (Minutes)	Reference
RT-LAMP	RNA	4000 copies/mL; 4 PFU/mL	~60	[36]
	RNA	~1 genome	~30	[37]
	RNA	−2.98 log_10_ PFU of ZIKV (~1/1000 PFU)	20	[13]
	RNA	1 aM synthetic ZIKV oligonucleotide	27	[38]
	RNA	14.5 TCID50/mL in RNA in serum and urine samples; 10 copies per reaction	15	[39]
	RNA	<100 PFU/mL	10–15	[35]
	RNA	35 copies of viral RNA per reaction (DENV 4) (10^4^ copies of RNA or 1.34 PFU).	50	[33]
	RNA	100 copies for DENV-1 and DENV-2; 10 copies for DENV-3 and DENV-4.	20–25	[40]
	RNA	10 RNA copies	30–45	[32]
	RNA	0.29 PFU/mL	~60	[41]
	RNA	19 PFU/mL	30	[42]
	RNA	2.57 copies/µL for JEV I and 2.34 copies/µL for JEV III	50	[43]
	RNA	5 pg of RNA	70	[44]
	RNA	12 copies/µl	60	[45]
	RNA	0.1 PFU/mL	30	[46]
CRISPR	ssRNA	2 aM	~30–180	[48]
	ssRNA	2 aM	60	[49]
	RNA	0.9 aM	<2 h	[50]
Microfluidic	NS1 protein	46.7 ng mL^−1^	20–60	[60]
	NS1 protein	10 ng/mL	<8	[58]
	Viral lysate	10^2^ particles/µL	<60	[61]
	NS1 protein	84.66 ng/mL	2	[62]
	RNA	10 PFU/ml	~180	[63]
	RNA	10^2^ copies/mL	40	[12]
	RNA	1 copy/µL	15	[64]
	RNA	1.56 × 10^5^ PFU/mL	35	[65]
	RNA	6 × 10^3^ PFU/mL	100	[66]
Nanotechnology-LSPR	NS1 protein	150 ng/mL	-	[72]
	NS1 protein	0.5 ng/mL	~45	[73]
	RNA	10^1^ TCID_50_ units	5	[74]
	Viral particle	10^5^ PFU/ml	1440 (24 h)	[75]
	Viral particle	10^7^ TCID_50_	<5	[76]
	NS1 protein	0.07 µg/mL	60	[77]
SERS	ssDNA viral	~6 aM	~120	[81]
	DNA	10 pM	~60	[82]
	Protein E	~5 fg/ml	~60	[83]
	Protein E	10 PFU/mL	<30	[84]

## Data Availability

All studies from which data are presented are cited and referenced in the manuscript.
